# An ipRGC-influenced/Non-Visual Spectral Occupant Model for lighting design, Part 2: Photobiological model implementation

**DOI:** 10.1177/14771535251368380

**Published:** 2025-10-14

**Authors:** J Alstan Jakubiec, A Alight

**Affiliations:** a John H. Daniels Faculty of Architecture, Landscape and Design, University of Toronto, Toronto, ON, Canada; b School of the Environment, University of Toronto, Toronto, ON, Canada

## Abstract

This paper pilots a method for the assessment of non-visual lighting effects based upon annual full-spectrum lighting calculations termed an ipRGC-influenced/Non-Visual Spectral Occupant Model (iNSOM). iNSOM calculates annualized melanopic irradiance, described in our previous paper, and derives seasonal and time-of-day metrics based on a collection of photobiological models from Postnova *et al*., Abeysuria *et al*. and Tekieh *et al*. to predict circadian dynamics, alertness and melatonin levels due to light exposure. Quantitative outputs of these metrics and novel spatial visualizations are then used to evaluate lighting design based on the predicted intrinsically photosensitive retinal ganglion cell (ipRGC)-influenced effect on occupants. The model is demonstrated using an example hospital ward model and tested under three daylight, electric light and screen device operational scenarios and two types of sleep quality. A comparative analysis between iNSOM and existing ipRGC-influenced lighting design metrics and standards demonstrates how ipRGC-influenced alertness and health metrics differ from existing saturation-based ipRGC-influenced lighting metrics.

## 1. Introduction

Human physiological responses to light are described as intrinsically photosensitive retinal ganglion cell (ipRGC)-influenced. ipRGCs are sensitive to shorter wavelengths of light than the visual system and because their impacts are on physiological factors other than visual perception.^1–3^ ipRGC-influenced effects of light influence alertness throughout the day,^4^ the onset of sleepiness^5^ and the timing of metabolic activity.^6,7^ ipRGCs also moderate short-term effects – neurophysiological stimulation^4^ and suppression of the sleep hormone melatonin^2,6^ for example. Several measures of non-visual lighting have emerged within architectural building simulation and performance analysis workflows^8–10^; however, currently it is challenging for designers to capture the contributions of light to instantaneous and daily physiological symptoms which are sensitive to spectral irradiance, the history of light exposure, timing and homeostatic body rhythms in a single workflow.

Part 1 of this series described a light simulation method from multi-spectral lighting simulations to time-series annualized melanopic irradiance – including daylighting, dynamic shades, electric lighting and its controls and the use of self-luminous screen devices. This paper describes the second part of a pilot framework that connects annualized melanopic irradiance simulations to physiologically based outcomes of ipRGC-influenced light exposure by implementing a combined photobiological model based on medical literature. We propose a framework for simulating and evaluating the photobiological outputs of ipRGC-influenced sleepiness, arousal and melatonin models and evaluate the outcomes using a series of different lighting design strategies involving both daylight and electric light exposure as well as sleep quality types. To the best of our knowledge, the ipRGC-influenced/Non-Visual Spectral Occupant Model (iNSOM) presented in this paper is the first to predict explicit biological effects of the resulting combined light spectrum over time in dynamically lit architectural spaces. In conclusion, we benchmark iNSOM’s results against existing metrics for ipRGC-influenced lighting design which are lighting threshold- and saturation-based. Important metrics and measures used in the text and analysis are defined in Appendix A for reference while reading this manuscript.

## 2. Previous work

### 2.1 ipRGC-influenced light evaluation metrics

Previous work to develop a framework for the ipRGC-influenced effects of lighting tends to utilize a threshold-based approach. Pechacek *et al*.^11^ is the earliest paper to develop a ipRGC-influenced health design framework. Using Radiance and Daysim, a Daylight Autonomy value was determined for a single viewpoint based on a vertical daylight illuminance threshold of 190 photopic lux.

Andersen *et al*.^12^ and Mardaljevic *et al*.^13^ divided the day into three distinct periods to relate to changing ‘non-visual effects’ (N-VE) depending on exposure time^4,14^: ‘circadian resetting’ from 6.00 to 10.00, ‘alerting’ from 10.00 to 18.00 and ‘light avoidance’ from 18.00 to 6.00. A 100% probability of a N-VE is when D55 equivalent illuminance ≥960 lx, and a 0% probability of an ipRGC-influenced effect occurs when D55 equivalent illuminance ≤210 lx. Between these two thresholds, the percent probability of a N-VE is interpolated by 100 × *E*_D55_/750 − 28. Andersen *et al*.^12^ introduced a novel visualization technique to summarize circadian potential (CP) for each period of the day. CP is the mean N-VE probability within a time-of-day period throughout a year.

Konis^10^ used equivalent melanopic lux (EML) >200 lx to indicate the extent to which the WELL standard, as defined at the time of the paper, was met by daylight, calling the result circadian frequency (CF). A later version of the WELL standard for workplaces differed from Konis’^10^ paper – divided into two tiers and using EML. The lowest minimum illuminance in an office space is at least EML > 150 lx ( $E_{v,\;\mathrm{mel}}^{D65}>136\;\mathrm{lx}$ or *E*_e,mel_ > 0.17 W m^−2^) at all workstations for at least 4 h (beginning at noon at the latest) at a height of 457 mm (or 18 inches) above the workplane in regularly occupied spaces. To meet WELL’s second level of certification is similar but with an EML >250 lx ( $E_{v,\;\mathrm{mel}}^{D65}>227\;\mathrm{lx}$ or *E*_e,mel_ > 0.30 W m^−2^) threshold.^15^ In residential buildings, WELL requires that light levels are dimmable, automated lighting must be dimmed after 8.00 pm, and workplane illuminance must meet EML < 50 lx ( $E_{v,\;\mathrm{mel}}^{D65}<45\;\mathrm{lx}$ or *E*_e,mel_ < 0.06 W $m^{-2}).$ ^16^ These threshold approaches do not communicate the resulting physiological effects due to the timing and intensity of light exposure.

Amundadottir *et al*.^17^ developed a cumulative daily threshold for ‘non-visual health potential’, known as non-visual direct response (nvRD). nvRD targets a daily value of 4.2 based on receiving vertical eye illuminance of 824 lx under a CIE D65 spectrum for a period of 5 h and is calculated using irradiance at 6-min time intervals.

Following the Second International Workshop on Circadian and Neurophysiological Photometry in 2019, expert consensus-based recommendations for light exposure were made.^18^ The recommendations, based on melanopic equivalent daylight illuminance (melanopic EDI, $E_{v,\;\mathrm{mel}}^{D65}$ ) include a minimum of $E_{v,\;\mathrm{mel}}^{D65}>250\;\mathrm{lx}$ (*E*_e,mel_ > 0.33 W m^−2^) vertical illuminance at eye-level when seated throughout the daytime. Beginning a minimum of 3 h before bedtime or during the nighttime when engaging in activities that require vision, melanopic EDI should not exceed $E_{v,\;\mathrm{mel}}^{D65}<10\;\mathrm{lx}\;$ (*E*_e,mel_ < 0.013 W m^−2^) vertical illuminance at eye-level when seated is recommended. The sleeping environment should be as dark as possible or not exceed $E_{v,\;\mathrm{mel}}^{D65}<1\;\mathrm{lx}$ (*E*_e,mel_ < 0.0013 W m^−2^).

ipRGC-influenced lighting has many physiological effects that independently respond to light at different times, exposure histories and durations. Thus, a single ipRGC-influenced equivalent value (i.e. EML, nvRD, CF, CP, etc.) may not capture the full gamut of physiological effects of light on people.

### 2.2 Photobiological effects models

Based on controlled experiments, several photobiological-driven models have been produced to assess physiological ipRGC-influenced effects of light exposure^1,19–21^ as well as the short-term alerting effects of light exposure.^3,4^ The Postnova *et al*.^1^ model, which is based on a variety of previous work,^19,20^ is the most recent model which predicts subjective sleepiness, reaction time and circadian phase shift. The Postnova model has been updated by Abeysuriya *et al*.,^2^ adding blood melatonin concentration and suppression due to short-term ipRGC-influenced light exposure. It was further modified by Tekieh *et al*.,^3^ who converted the light input units from photopic illuminance (recorded from a specific 4100 K fluorescent lamp) to melanopic irradiance^22^ and added the instantaneous alerting effects of light on sleepiness. These frameworks require a time-varying input of melanopic irradiance and optionally sleep–wake state. They predict biological rhythms and timing but do not describe a workflow for simulating melanopic irradiance in a space, assessing the physiological model outputs, nor for visualizing ipRGC-influenced outputs during a design process.

Although most measures of ipRGC-influenced lighting discussed within this paper rely upon the response of the photopigment melanopsin, melanopsin is only the primary determinant in the firing rate of ipRGC’s.^23^ Rod and cone photoreceptors also play a mediating role in instantaneous and long-term ipRGC-influenced effects which is still being understood.^24–27^

## 3. Method

### 3.1 Geometric model, spectral irradiance calculations and light scenarios

**Figure 1:**
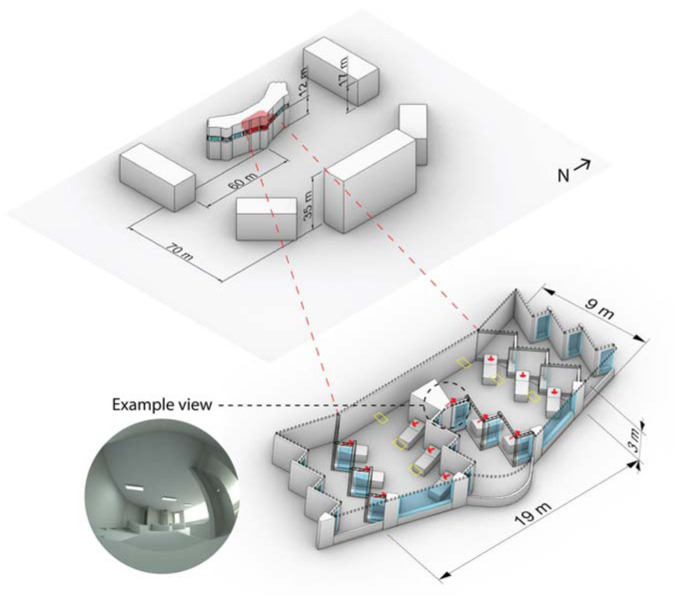
Hospital ward model used for lighting calculations with view directions indicated by red arrows, ambient luminaires indicated by yellow rectangles, and an example view circled

**Figure 2:**
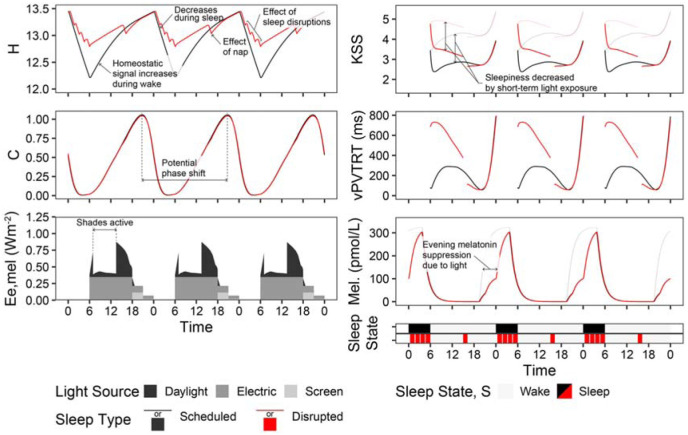
Photobiological dynamics of the combined time-spectrum-photobiology model shown for the example view in the dimming scenario. Light grey or red lines indicate simulated outcomes in the absence of light

**Figure 3:**
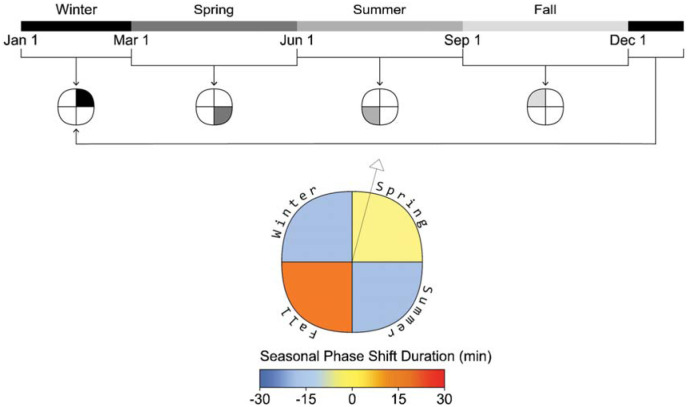
Phase shift plot example and seasonal timeline (example view, daylight lighting, scheduled sleep)

**Figure 4:**
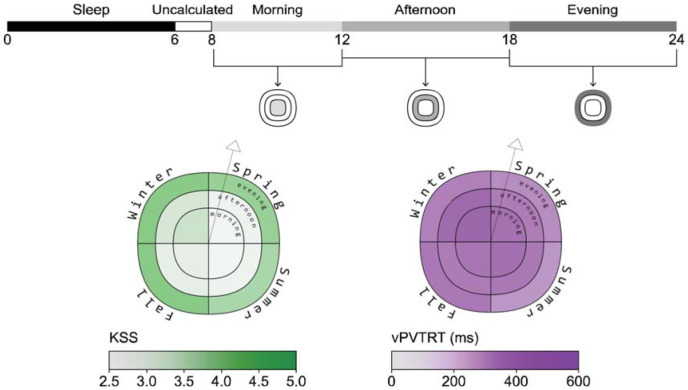
Sleepiness and alertness plots example (example view, daylight scenario, scheduled sleep)

**Figure 5:**
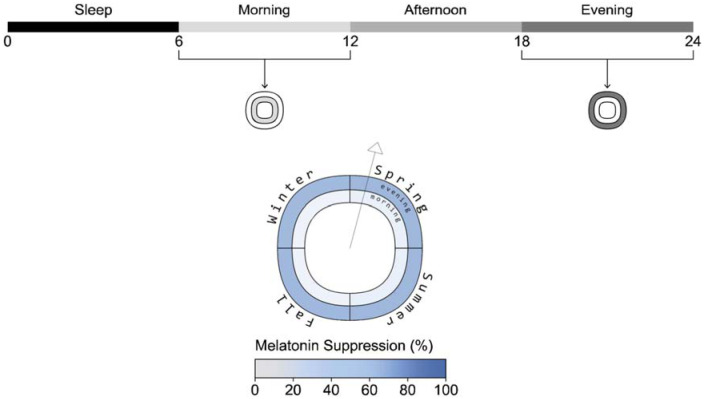
Melatonin suppression plot example (example view, electric lighting scenario, scheduled sleep)

A well-daylit 12-person, east-facing ward with spectrally neutral materials based on the Ng Teng Fong hospital is used for analysis. The 3D model, as simulated with a moderate surrounding urban context, is illustrated in Figure 1. We simulated a patient view from each of the 12 beds in the ward reclined at a 30° angle and indicated by the red arrows and example rendering in Figure 1. The simulation model, material properties, methods and time-varying irradiance results are presented in the first part to this paper. Dynamic shades are lowered when any viewpoint’s vertical eye illuminance would exceed 3000 lx without the prescence of shades. Generally, there is a short spike of daylight in the morning before shades lower until around 1 pm when received daylight illuminance is at its peak and gradually declines until sunset. The indicated example view will be used for reference later in this manuscript (Figures 2 to 5).

To demonstrate iNSOM, we apply a set of electric lighting and screen use scenarios combined with annual daylight access. While there are a large variety of screen devices which a person might use, we chose values which represent a bright LED screen at a typical viewing distance with a daytime (6500 K) and nighttime (1900 K) mode. The scenarios we apply are described below and are presented with greater fidelity in Part 1 of this manuscript alongside spectral descriptions of the light sources and material properties.

(1) *Daylight only (daylight)*– Light exposure is only due to daylight.
(2) *Daylight with constant electric light and monitor screen spectra (electric light)*– Daylight as per the daylight only scenario is present. Electric lighting with a correlated colour temperature (CCT) of 6500 K is turned on during all waking hours (6 am to midnight). Monitor screens with a blue enriched screen with a CCT of 6500 K are used throughout the evening (6 pm to midnight).
(3) *Daylight, colour-changing electric lighting and evening monitor screen spectra (dimming)*– Daylight is present as per scenarios 1 and 2. Electric light begins as per scenario 2 but shifts to a warm 2800 K spectrum from 6 pm to 10 pm in the evening and is dimmed to half power from 10 pm to midnight. The monitor screen spectra shifts to a blue-depleted 1900 K CCT from 9 pm to midnight to reduce ipRGC-influenced effects.

### 3.2 Photobiological effects model

We implement a dynamic photobiological model of sleep/wake dynamics and cognitive performance based on a lineage of biological modelling work (described in Section 1) including Postnova *et al*.,^1^ Abeysuriya *et al*.^2^ and Tekieh *et al*.^3^ The basis of these models is complimentary homeostatic (*H*) and circadian (*C*) drives.^1^ The homeostatic system’s need for rest is increased during wake and the consequent utilization of wake-active neurons, and it decreases under rest during sleep. As *H* increases, it activates a sleep drive and sleep-active neurons. *C* is periodic across an approximately 24-h period and is modulated by sleep and entrainment history as well as transient light exposure, and it inhibits the sleep drive and sleep-active neurons. Natural sleepiness occurs when *H* increases to the point that it surpasses the impact of *C* to wakefulness. These interactions are modelled by a series of 6 differential equations (with respect to time) and 11 further complementary equations determining aspects such a sleep/wake state, circadian phase and photic- and nonphotic-drives to the circadian system; they are described in detail by Postnova *et al*.^1^ Alertness predictions such as subjective sleepiness (Karolinska Sleepiness Scale, KSS) and reaction time (visual Performance Vigilance Test Reaction Time, vPVTRT) are determined based on the relative values of *H* and *C* during wake and the short-term effects of light exposure.

We also implement Abeysuriya *et al*.’s model extension^2^ of melatonin synthesis, light-based melatonin inhibition and resulting melatonin blood plasma concentration in combination with Tekieh *et al*.’s^3^ conversion of the model to SI units of melanopic irradiance (*E*_e,mel_, W m^−2^). The extension to assess melatonin blood plasma concentration relies on a model of melatonin secretion using two differential equations based on Postnova’s circadian drive paired with an instantaneous melanopic irradiance-based inhibition function. Using the combination of the Postnova, Abeysuriya and Tekieh models, we predict subjective sleepiness, mean reaction time, melatonin blood plasma concentration and circadian phase shifting. The model equations and starting values are reported in Supplemental Material.

A more detailed description of the process for obtaining melanopic irradiance data can be found in Part 1 of this series. In summary, we produced a Python library to process time-varying spectral irradiance, convert results into melanopic irradiance (*E*_e,mel_, W m^−2^),^22^ interpolate the melanopic irradiance into an equally spaced annual timeseries, implement the photobiological models described above across an entire year and post-process the results to generate relevant seasonal and time-of-day metrics which are described in this manuscript. This library, along with our simulation data, is published online and linked as Supplemental Data.

Some specifics of our implementation follow: The differential equations for the photobiological model are run at a time interval of 20 s. In the implementation of the model, sleep can be allowed to occur naturally (when the mean voltage of the wake-active neurons falls below a threshold^1^) or, as is more common, on a schedule. We simulate a regular, ‘scheduled’ sleep type and a ‘disrupted’ sleep type based on meta-analysis of sleep quality in hospital patients.^28,29^ In the scheduled sleep pattern, we implement a sleep schedule from midnight until 6.00 am each day with wake from 6.00 am until midnight. The scheduled sleep type represents a moderately poorly slept person with extremely good sleep habits. The disrupted sleep type lays down at the same time (midnight), but sleep onset is delayed for 24-min (12.24 am sleep start). Sleep proceeds for 5.6 h with 3, 12-min interruptions during the sleep period as might occur in a care setting. A 1.2-h midday nap occurs from 3.00 pm until 4.20 pm. Before a virtual person’s photobiological response is assessed, they are computationally entrained to the sleep schedules described above, either scheduled or disrupted, for a period of seven days. Once entrained on this typical schedule, we run simulations for an entire calendar year based on the framework described in Part 1 for the electric lighting and monitor use indicated for our three scenarios (daylight, electric light and dimming).

As described in the preceding sections and paragraphs, variations in electric lighting control systems, monitor screen use and some aspects of user behaviour (such as dynamic window shade use, screen device use and sleep types)^30^ are thus accounted for and their impacts assessed. Following this, age (corneal transmission) and other personal factors could also be adapted into the model. It is not possible that our iNSOM model will reproduce light exposure and ipRGC-relevant behaviour exactly as it would occur in the real world; however, we contend that enough relevant parameters can be included such that the results are appropriate to assess daylighting and electric lighting controls in the design of spaces with long-term occupants.

Figure 2 illustrates the dynamics of the photobiological model over three days (from September 21 to September 23) of the annual simulation period from the viewpoint indicated in Figure 1 and the two sleep schedules. *H* and *C* depict the aforementioned homeostatic and circadian drives where increasing *H* drives sleepiness and increasing *C* drives wakefulness. State illustrates the two sleep–wake schedules we have assigned – scheduled (black) and disrupted (red). *E*_e,mel_ displays the time-series melanopic irradiance due to daylight, electric light exposure and monitor screen use. *E*_e,mel_ influences *C* and all output metrics (KSS, vPVTRT and Mel.) except for during periods of sleep. Daily phase shifting is calculated based on the change in the time of peak melatonin blood plasma concentration from one day to a subsequent day, and the difference in *C* between two days, the circadian system phase, can also illustrate this shift. In this figure, electric light and screen-source irradiance are present during the day, and both are dimmed during the evening. KSS and vPVTRT show predicted subjective and objective performance – sleepiness and mean reaction time (ms) respectively. In the case of the KSS plot, higher numbers indicate increased sleepiness – 1 represents an extremely alert state, and 9 is fighting sleep. According to Postnova *et al*.,^1^ both KSS and vPVTRT are not valid for the first 2 h after waking; therefore, they are excluded from statistical analysis later in this manuscript during those time periods. Finally, Mel. displays the varying concentrations of melatonin present in blood plasma.

Figure 2 also illustrates how much sleep quality and the ability to have uninterrupted sleep can influence the resulting metrics. With three sleep interruptions per evening, the *H* drive towards sleep is always much greater for the disrupted sleep type than for the scheduled sleep type. As a result, KSS illustrates significantly greater feelings of sleepiness during the day, and vPVTRT illustrates significantly higher reaction times during the day. This illustrates the need for caregiving spaces to encourage productive sleep and reduce sleep disruptions, of which light during the evening and night is a part. The scheduled and disrupted sleep types along with the three lighting scenarios are used for analysis throughout this manuscript.

### 3.3 Photobiologically derived metrics

As described in the introduction to this manuscript, most photobiological architectural lighting metrics are frequency or saturation-based and indicate the amount of light required for generalized ipRGC-influenced effects either calculated based on the percentage of hours in a year^10^ or cumulative effect per day.^12,13^ When assessing photobiological effects, the timing of light exposure, sleep history and light history are crucial. We generate seasonal metrics over the course of a typical year based on their meteorological dates; spring (March 1 to May 31), summer (June 1 to August 31), fall (September 1 to November 30) and winter (December 1 to February 28). To spatially display the metrics, we developed a visualization tool that utilizes a similar technique to that implemented by Andersen *et al*.^16^ and Mardaljevic *et al*.^17^ who used a circular figure with pie-like divisions and concentric bands at each simulated occupant location. Their bands are divided to indicate different times of day, and the circle is divided into radial slices that indicate view direction. However, our plot visualizes data for a single view location and direction, illustrated by a vector arrow, and the quadrants of the plot represent seasons rather than view directions (see Figure 3). This allows a reader to evaluate seasonal changes in ipRGC-influenced light exposure and predicted physiological outcomes. An example display is presented alongside each photobiologically derived metric described below in Figures 3 to 5.

*Phase shifting*: Daily phase shifting is calculated based on the change in the time of peak melatonin blood plasma concentration from one day to a subsequent day, or a cumulative phase shift can be calculated over a longer period. We present the cumulative phase shift over each meteorological season to display the seasonal influence of light exposure on circadian rhythms, and shorter durations of exposure to the same daily regime would reduce the magnitude of the reported impact. Figure 3 illustrates the plot to display the cumulative phase shift for an occupant at a fixed viewpoint over each season of the year in minutes and is displayed for the daylight scenario with scheduled sleep.

*Melanopic irradiance and alertness*: Postnova *et al*.^1^ indicated several alertness measures based on a regression between *H*, *C* and measured study data. We illustrate two herein: predicted sleepiness rated by the KSS^29^ and the predicted mean reaction time on a visual performance vigilance test (vPVTRT). KSS evaluates in a range from 1 (extremely alert) to 9 (extremely sleepy, fighting sleep), and the median point on the KSS scale is 5 (neither alert nor sleepy). The implementation of predicted KSS considers the instantaneous alerting effects of light exposure,^3^ but these effects have not yet been identified for vPVTRT. The selection of these two metrics can therefore be understood as separating the influence of long-term ipRGC-influenced circadian effects (vPVTRT) versus the influence of the short-term influencing effects of light (KSS).

KSS and vPVTRT are presented as mean values over three distinct periods in Figure 4: morning (8.00 to 12.00), afternoon (12.00 to 18.00) and evening (18.00 to 24.00). Figure 4 shows results for the daylight scenario with scheduled sleep. For each season:

- The inner circle displays mean alertness during the morning period.
- The mean afternoon alertness is presented in the middle ring.
- The outer ring represents the mean alertness during the evening period.

Sleepiness and reaction time is not reliably calculated by the photobiological model within two hours of wake (although shown in Figure 2)^1^; therefore, the morning alertness period starts at 8 am which is 2 h after waking at 6 am.

*Melatonin suppression*: Melatonin blood plasma concentrations in pmol L^−1^ are calculated based on the driving forces for the circadian system, *C* and short-term light exposure which suppresses melatonin synthesis.^2,3^ Melatonin suppression is calculated as the difference between the integral of daily melatonin blood plasma concentration without light exposure, the maximum possible, compared to an actual day under light exposure using Equation (1).^3^ AUC_day_ stands for area under the curve and is the integral of a day’s blood plasma melatonin curve as displayed in Figure 5, and AUC_dark_ is the integral over the same period but bereft of the effect of light exposure on suppressing melatonin.

(1) $$\mathrm{Supression}=100.\frac{\left(\mathrm{AU}C_{\mathrm{dark}}-\mathrm{AU}C_{\mathrm{day}}\right)}{\mathrm{AU}C_{\mathrm{dark}}}$$

We separate and display melatonin suppression into morning (8 am to noon) and evening (6 pm to midnight) periods. The body does not secrete melatonin during the afternoon, so showcasing this period would be redundant as there is little melatonin in the bloodstream to be suppressed by light exposure for a normally entrained human. The melatonin suppression plot (Figure 5) shows the percentage of melatonin suppression over each season for two periods:

- The inner ring represents the mean percentage of morning melatonin suppression for each season.
- The mean percentage of evening melatonin suppression is displayed in the outer ring for each season.

The data shown in the figure is from the electric lighting scenario with scheduled sleep.

## 4. Results

### 4.1 Demonstration of iNSOM

**Figure 6:**
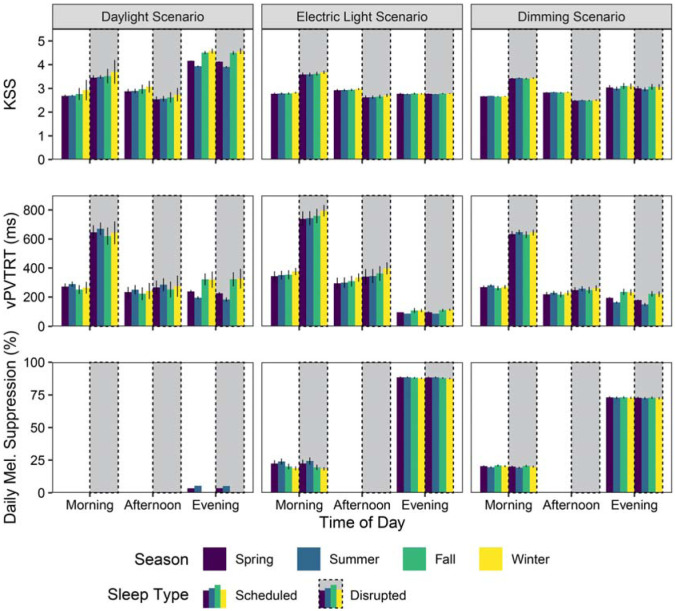
iNSOM results for melanopic irradiance, KSS, reaction time and melatonin suppression across all, scenarios, daily time periods and seasons

**Figure 7:**
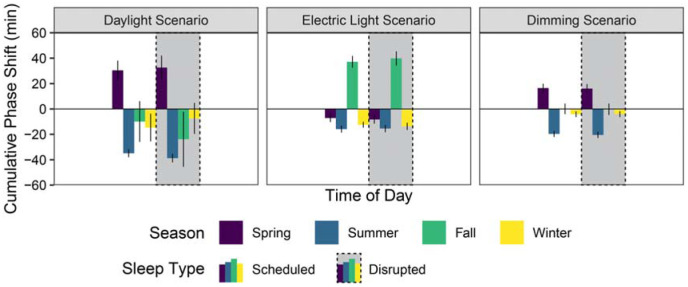
iNSOM mean phase shift results across all scenarios and seasons

Figure 6 illustrates the mean photobiological results of the 12 views in Figure 1 for each scenario, sleep schedule type and time period. Colours indicate the four seasonal temporal divisions: spring is purple; summer is blue; fall is green and winter is yellow. Vertical bars indicate ±1 SD across the 12 spatial results, indicating how much occupant position in the space influences the physiologically predicted result. Results are further separated by the three lighting scenarios (major column organization), sleep type (presence of dashed boxes) and three daily time periods (*x*-axis ticks). Results for the scheduled sleep type are shown. Stemming from the melanopic irradiance quantities simulated in Part 1 of this manuscript, mean KSS, mean vPVTRT (ms) and mean melatonin suppression (%) are represented in the figure. Circadian phase shifting is represented separately in Figure 7 as it is not calculated by time of day but cumulatively across a season. Quantitative visualizations such as this express discrete effects on space participants within a design and how much these predicted ipRGC-influenced measures vary by season, time of day and spatially across that design.

KSS is easily saturated by instantaneous light exposure^3^; therefore, it is relatively low throughout the morning period for the scheduled sleep type (6 hours of regular sleep per day) with a mean KSS of 2.7 across all seasons, scenarios and views. However, sleep quality plays a mediating role in this effect as the same mean value is 3.5 when applied to the disrupted sleep type results. Afternoon values exhibit moderate and flipped differences with a mean KSS of 2.9 for scheduled sleep and 2.6 for disrupted sleep, showing the ability of the short nap to address sleep deficiencies. The only significant KSS differences between lighting scenarios are observed during the evening period when there is less daylight present. Relatively small shifts in evening melanopic irradiance from artificial light sources results in large differences in KSS between scenarios across both sleep types for the evening period such that the daylight scenario has the greatest evening subjective sleepiness at a mean KSS of 4.3 and the electric light scenario has the lowest evening subjective sleepiness at a KSS of 2.8. In relative terms, a KSS of 4.3 is between ‘rather alert’ and ‘neither alert nor sleepy’ while 2.8 is near ‘alert’.^31^ Contrary to expectations, there were no predicted differences in KSS during the evening period between scheduled and disrupted sleep types.

For the scheduled sleep type, predicted mean vPVTRT reaction times during the evening period were the fastest (scenario, spatial and seasonal mean of 191 ms) compared to the morning period (298 ms) and afternoon period (256 ms). For both sleep types, reaction time was fastest in the evening for the electric lighting scenario, illustrating the significant alerting effects of strong evening light (460% faster than the morning period and 340% faster than the afternoon period based on mean values combining space and sleep type). The morning period’s reaction time was slowest for the electric light scenario compared to the other two – for the scheduled sleep type morning reaction times were 56% slower than the evening, and for disrupted sleep this increases to 268%. Overall, disrupted sleep significantly increases reaction time during the morning compared to the rested sleep type (an 128% increase based on means across space, seasons and lighting scenarios). This difference is most apparent during the electric light scenario where strong evening light increases the mean reaction time across seasons to 759 ms for the disrupted sleep type (an 18% increase over the daylight scenario and a 19% increase over the dimming scenario).

Melatonin suppression is highest in the evening across all scenarios as humans are most sensitive to melatonin suppression in the evening when natural melatonin secretion is the highest, and it does not display any discernible differences across sleep types. The electric light scenario results in the most extreme suppression, with a seasonal-spatial mean of 88.1% suppression in the evening, and a seasonal-spatial mean of 21.2% suppression in the morning. The daylight scenario demonstrated the least suppression with a seasonal-spatial mean of 2.5% evening suppression, and seasonal-spatial mean of 0.1% morning suppression. This is compared to seasonal-spatial means of 72.8% evening suppression and 20.1% morning suppression in the dimming scenario.

Phase shifting (displayed in Figure 7) is highly sensitive to seasonal light exposure changes, but it is not strongly influenced by sleep type; therefore, this paragraph analyses both sleep types together when considering mean values. Summer in all three scenarios produced the strongest phase advance with longer day lengths and later sunsets coupled with significantly shade-reduced morning daylight (mean of −24.2 min across all results). The daylight, electric light and dimming scenarios respectively produced a −36.9 min, −15.7 min and −20.1 min phase advance in the summer. Conversely, winter shows the shortest phase change with minimal morning and evening sun exposure (mean advance of −9.0 min across all results). The fall season in the electric light scenario experienced the largest phase delay (38.5 min) of any season and scenario.

### 4.2 Spatial analysis

**Figure 8:**
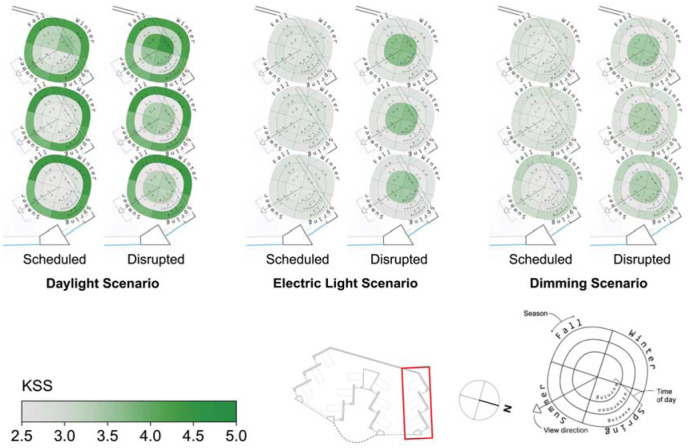
Spatial visualization plots of KSS for three scenarios and two sleep scenarios in the North side of the space

**Figure 9:**
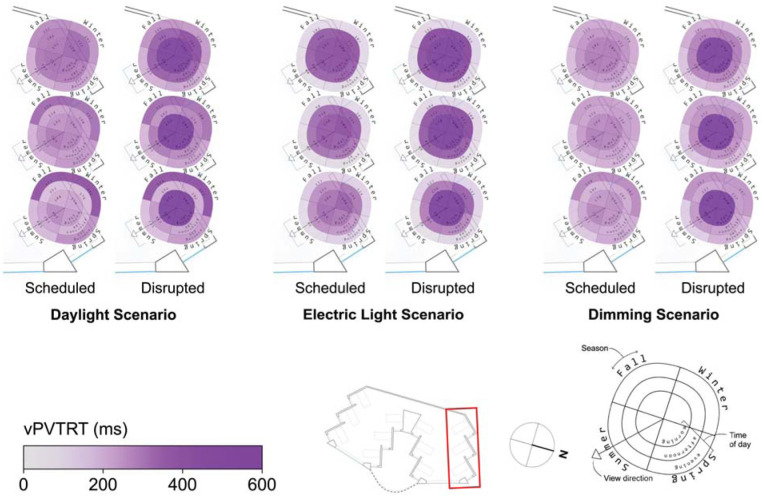
Spatial visualization plots of vPVTRT for three scenarios and two sleep scenarios in the North side of the space

**Figure 10:**
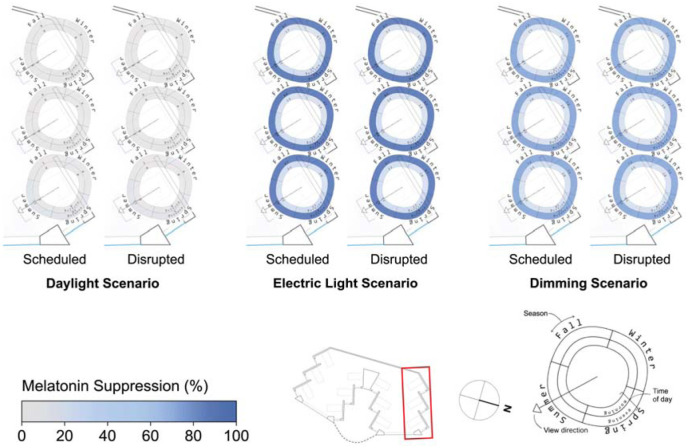
Spatial visualization plots of melatonin suppression for three scenarios and two sleep scenarios in the North side of the space

**Figure 11:**
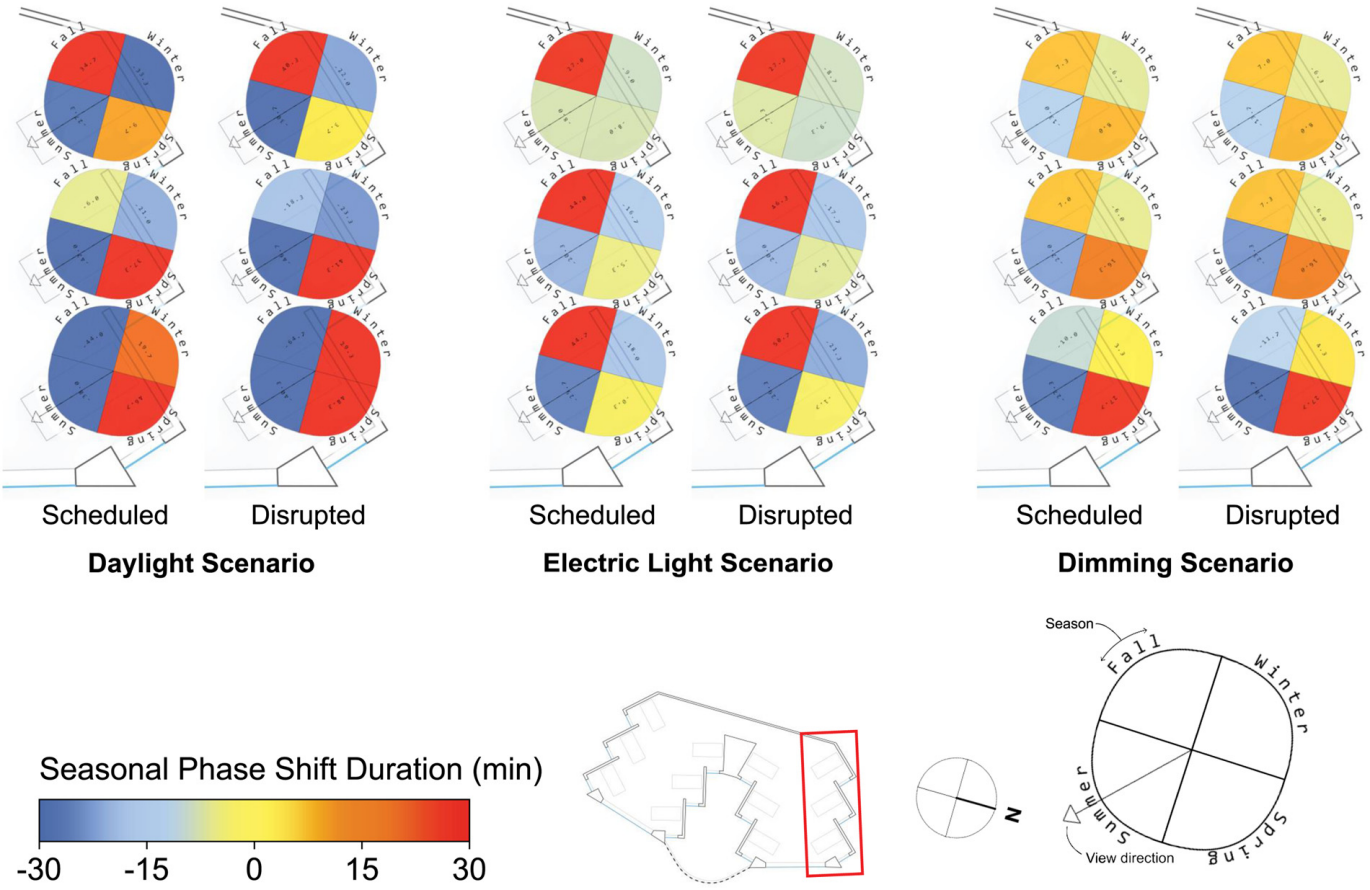
Spatial visualization plots of seasonal phase shifting for three scenarios and two sleep scenarios in the North side of the space

The spatial distribution of the results depicted in Figures 6 and 7 are illustrated in Figures 8 to 11 to assess the potential for spatial differentiation in ipRGC-influenced lighting effects in a well-daylit space. For conciseness and to enable the viewing of results for both sleep types, only the North quarter of views (three) of the hospital ward are shown, but any statistical analysis encompasses all 12 views. In each plot, results of the three lighting scenarios are presented from left to right. In each scenario, three scheduled and three disrupted sleep type results are shown from the front (window-side) to the back of the space. Figures 3 to 5 provide context and explanation for the reading of each diagram.

The effect of daylight on reducing sleepiness, KSS, can be observed in Figure 8. The alerting effects of daylight are observed for the daylight scenario’s views at the front of the space (all-season mean of 2.6 for scheduled sleep and 3.4 for disrupted sleep) compared to those at the back of the space (all-season mean of 3.0 for scheduled and 3.8 for disrupted sleep). Daylight scenario views at the back of the space are particularly sleepier during winter (mean KSS of 4.3 for the disrupted sleep schedule). These spatial differences in KSS are largely eliminated within scenarios once electric lighting is introduced. In the electric lighting scenario, the maximum KSS varies from the mean by only 3.1% for the scheduled sleep type and 2.4% for the disrupted sleep type, and similar results are found for the dimming scenario.

Spatial and seasonal visualizations of reaction time (vPVTRT) are shown in Figure 9, and more spatial variation is found compared to KSS as more light seems to be required to affect vPVTRT. This may be because vPVTRT in the current photobiological model^1–3^ is a function of *C* and *H* alone and does not include an instantaneous alerting factor yet like KSS. Nonetheless, as the viewpoints move away from the window and towards the back wall, the plots represent a slower reaction time during the morning and afternoon times for all scenarios following reduced daylight exposure’s effect on the circadian drive, *C*. For example, the front, middle and back views in the dimming scenario during the afternoon time period illustrate mean vPVTRT of 217 ms, 239 ms and 258 ms, respectively. During the evening time period, this effect does not hold as there is relatively little daylight, and electric light is evenly distributed throughout the space.

Melatonin suppression varies little spatially between views and sleep types as can be observed in Figure 10 for all scenarios. The maximal variance for melatonin suppression is for the daylight scenario where views at the front of the space experience evening melatonin suppression of 6.1% where views in the back experience only 0.1% (based on means across both sleep types). The electric lighting and dimming scenario plots indicate similar levels of suppression as viewpoints move away from the window. Melatonin suppression varies the most between lighting scenarios – it is minimal in the daylight only scenario, highest in the electric lighting scenario and lowest in the dimming scenario as presented in the discussion surrounding Figure 6.

Of all the ipRGC-influenced effects, phase shifting (Figure 11) demonstrates the greatest spatial variation and also demonstrates variation between sleep types, especially for the daylight scenario. Views in the front of the spaces with greater light avoid phase delays in winter for the daylight scenario across both sleep types (mean phase shift of 24.1 min in the front compared to −27.1 min in the back). During summer, daylight scenario phase shifting does not vary spatially. During the spring and fall, daylight scenario phase advances are alleviated or delays are mitigated by greater exposure to light at the front of the space. These relatively extreme spatial effects are still present but significantly reduced in the electric lighting and dimming scenarios with more controlled light levels throughout waking hours. For example, fall mean phase shift differences between the front and back views of 76.8 min, 19.7 min and 16.6 min can be observed for the daylight, electric light and dimming scenarios, respectively.

### 4.3 ipRGC-influenced health evaluation metric comparison

Using the spectral irradiance calculations from the method described in Part 1, we followed the models by Amundadottir *et al*.^12^ which calculates ‘non-visual health potential’ using the nvRD model, Mardaljevic *et al*.^17^ which predicts potential for an N-VE, and calculated the percentage of time which the WELL daytime standard^13^ of 250 EML was met. The current WELL standard^13^ does not include a specific maximum threshold for nighttime light levels on occupants’ eyes, only on the workplane; therefore, it was not included in this section. In implementing the WELL standard, we also did not consider the stipulation that electric light alone must be able to meet the thresholds without daylight. We also calculated the percentage of time the Brown *et al*. thresholds were met during daytime and nighttime hours. We calculated each method according to the time periods described in Section 3.3.

We used statistical correlation to determine how these existing ipRGC-influenced metrics relate to the photobiologically derived metrics described and piloted in this paper. Because the ipRGC-influenced metrics are continuous variables, Pearson correlations were used to compare the existing ipRGC-influenced metrics (mean nvRD, percentage of time WELL EML > 250 lx daytime standard met, percentage of time the Brown *et al*. recommendation met, resetting period N-VE, alerting period N-VE and avoidance period N-VE) with the iNSOM metrics. Correlations were derived separately for the scheduled and disrupted sleep types. As a result, each metric dataset contains 144 data points from each viewpoint (*n* = 12), lighting scenario (*n* = 3) and season (*n* = 4), and two sets of correlations are generated based on the iNSOM sleep types. The resulting Pearson coefficients are displayed in Tables 1 (scheduled sleep) and 2 (disrupted sleep). To describe the strength of association between ipRGC-influenced health metrics, effect size categorizations for Pearson coefficients (*r*) from Ferguson^32^ are followed during this discussion: not practically significant effect (*r* < 0.2), practically significant effect (0.2 < *r* < 0.5), moderate effect (0.5 < *r* < 0.8) and strong effect (*r* > 0.8).

**Table 1:** Pearson correlations between existing circadian lighting metrics and novel photobiologically derived metrics for ‘scheduled’ sleep behaviour

Metric	Metric’s time period	Phase shift	Melatonin suppression		KSS			vPVTRT		
			Morning	Evening	Morning	Afternoon	Evening	Morning	Afternoon	Evening
nvRD		0.09	0.74**	0.75**	−0.47**	−0.63**	−0.72**	0.12	−0.10	−0.46**
WELL	Daytime	0.09	0.79**	0.81**	−0.33**	−0.50**	−0.79**	0.23**	0.03	−0.54**
N-VE	Resetting	0.03	0.41**	0.38**	−0.32**	−0.45**	−0.32**	−0.21	−0.38**	−0.18*
	Alerting	0.01	0.49**	0.46**	−0.40**	−0.60**	−0.43**	−0.12	−0.34**	−0.31**
	Avoidance	−0.07	0.52**	0.62**	0.14	0.13	−0.62**	0.74**	0.60**	−0.83**
Brown *et al*.	Daytime	0.08	0.83**	0.88**	−0.23**	−0.36**	−0.85**	0.42**	0.22	−0.72**
	Nighttime	−0.10	−0.99**	−0.98**	0.06	0.20*	0.96**	−0.45**	−0.31**	0.67**

* *p* < 0.05. **p < 0.01.

**Table 2:** Pearson correlations between existing circadian lighting metrics and novel photobiologically derived metrics for ‘disrupted’ sleep behaviour

Metric	Metric’s time period	Phase shift	Melatonin suppression		KSS			vPVTRT		
			Morning	Evening	Morning	Afternoon	Evening	Morning	Afternoon	Evening
nvRD		0.08	0.74**	0.75**	−0.47**	−0.57**	−0.72**	−0.03	−0.12	−0.46**
WELL	Daytime	0.09	0.78**	0.81**	−0.32**	−0.42**	−0.79**	0.09	0.01	−0.55**
N-VE	Resetting	0.03	0.40**	0.38**	−0.36**	−0.48**	−0.32**	−0.33**	−0.40**	−0.22**
	Alerting	0.01	0.49**	0.46**	−0.46**	−0.58**	−0.43**	−0.27**	−0.37**	−0.34**
	Avoidance	−0.04	0.53**	0.61**	0.22**	0.17*	−0.59**	0.63**	0.57**	−0.80**
Brown *et al*.	Daytime	0.08	0.83**	0.88**	−0.20*	−0.29**	−0.85**	0.28**	0.18*	−0.71**
	Nighttime	−0.13	−0.98**	−0.98**	0.03	0.13	0.96**	−0.35**	−0.27**	0.67**

* *p* < 0.05. **p < 0.01.

The trends of correlation between existing metrics and photobiological predictions observed between Tables 1 and 2 follow the same patterns with a few exceptions. Notably for disrupted sleep, daytime light metrics have less impact on vPVTRT morning reaction time (WELL daytime of *r*_disrupted_ = 0.09, *p*_disrupted_ > 0.05 vs. *r*_scheduled_ = 0.23, *p*_scheduled_ < 0.01 and Brown *et al*. daytime of *r*_disrupted_ = 0.28, *p*_disrupted_ < 0.01 vs. *r*_scheduled_ = 0.42, *p*_scheduled_ < 0.01). This result shows some support for N-VE’s more fine-grained time periods as the N-VE resetting period has practically significant and meaningful correlations with morning vPVTRT for disrupted sleep (*r*_disrupted_ = −0.33, *p*_disrupted_ < 0.01) in a direction that is sensible – more light correlates with lower reaction time.

As seen in Tables 1 and 2, no metrics are correlated with phase shift, although it can be noted that in an earlier version of this work without urban context and dynamic shading, existing light exposure metrics correlated meaningfully with phase shifting.^33^ The only strong effects (*r* > 0.8) in the correlation table are found between the percentage of time the Brown *et al*. thresholds^15^ are met and melatonin suppression (Brown *et al*. daytime-morning melatonin suppression: *r*_scheduled_ = 0.83; Brown *et al*. nighttime-evening melatonin suppression: *r*_scheduled_ = −0.98), between the time the Brown *et al*. thresholds are met and evening KSS (*r*_scheduled_ = 0.96), between the time the WELL daytime standard is met and evening melatonin suppression (*r*_scheduled_ = 0.81) and between N-VE avoidance and evening reaction time (*r*_scheduled_ = −0.83). Again, the N-VE avoidance finding seems sensible in that higher evening lighting has a strong impact on reducing reaction time as well as alertness (KSS). That the meeting of the Brown *et al*. evening lighting thresholds correlate with decreased melatonin suppression and increased sleepiness is reassuring. These results highlight the importance of considering electric lights and evaluating evening light exposure separately from daytime because of their differing ipRGC-influenced effects.

Overall nvRD, a daily integral of light exposure, correlates with the reduction of sleepiness (KSS) as per its intent at a level between practically significant and moderate. Compliance time with WELL’s daytime light metric correlates with melatonin suppression (between the top end of moderate effect and the lower end of strong), KSS (between practically significant and moderate) and with evening vPVTRT (practically significant). N-VE correlates (within a range of practically significant to moderate) with metrics that are from the same time period. For example, alerting N-VE is correlated with afternoon KSS (*r*_scheduled_ = −0.60). Time complying with the Brown *et al*. daytime and nighttime thresholds correlates in the strong effect range with morning and evening melatonin, respectively (*r*_scheduled_ = 0.83 and *r*_scheduled_ = −0.98). Similarly, meeting the Brown *et al.* threshold for nighttime light exposure yields a strong correlation effect with evening sleepiness (KSS *r*_scheduled_ = 0.96) – the strongest correlated metric to evening KSS.

## 5. Discussion

### 5.1 ipRGC-influenced/Non-Visual Spectral Occupant Model

The implementation of iNSOM presented herein predicts physiological ipRGC-influenced effects from a set of lighting design and occupant parameters on an annualized basis. Each of the metrics responds differently to the intensity and timing of melanopic irradiance exposure which offers designers and researchers more nuanced information on their lighting design compared to existing frameworks that simplify symptoms of the non-visual system into a single metric. KSS and melatonin suppression are governed by a similar underlying non-visual biological circuit, but the range of melanopic irradiance they are sensitive to differs.^1,3^ KSS varies primarily in low melanopic irradiance conditions as the metric is quickly saturated by increased melanopic irradiance.^3^ The instantaneous effect of melanopic irradiance on KSS, for example, is almost fully saturated at *E*_e,mel_ = 0.075 W m^−2^ ( $E_{v,\mathrm{mel}}^{D65}=57\;\mathrm{lx}$ ) while *E*_e,mel_ = 1 W m^−2^ ( $E_{v,\mathrm{mel}}^{D65}=754\;\mathrm{lx}$ ) is required to achieve a 95% melatonin suppression effect.

There is also the matter of each metric’s time-dependent sensitivity. As described in the methods section, there is almost no melatonin circulating in the bloodstream in the early afternoon for an individual that sleeps during the night. This affects the ability of lighting design to influence melatonin suppression during the afternoon because there is no melatonin to suppress. Breaking down the ipRGC-influenced effect of lighting into individual effects prompts new questions regarding the overall applicability of single metrics created in previous methods. Since symptoms differ in various biological mechanisms’ response to light, the range of melanopic irradiance to which they are sensitive, and their baseline cycle, a single metric cannot represent the various ipRGC-influenced responses as demonstrated by the varying correlation strengths between predicted ipRGC-influenced responses and existing design metrics in Table 2.

Phase shifting is a particularly useful example of the importance of the timing of light. Light at night causes a phase delay, while light in the morning causes a phase advance. However, it is quite possible to experience both light at night and light in the morning. It is therefore helpful to understand circadian lighting design in terms of the net circadian effect on the occupant instead of relying on general advice about the timing of light. The cumulative effect of early morning and late evening light exposure can be demonstrated when analysed through a specific ipRGC-influenced effect such as phase shifting. In our results, the winter season experienced the strongest phase advance because there is the least amount of evening light. Although winter experiences the least melanopic irradiance in the morning compared to other seasonal periods, its shorter days still result in a net phase advance.

As demonstrated by phase shifting in winter, the absence of light at night is just as important as sufficient light in the morning. Although morning light can be achieved by designing a properly daylit building, light at night is an inherently artificial lighting issue. The scenarios in this study suggest that the harmful effects of light (as seen in the electric light scenario) can be reduced by strategic lighting schedules that utilize dimmer and warmer-colour light in the evening, demonstrated by the dimming scenario. The 24-h circadian health model is crucial in buildings that house shift workers because artificial lighting may be used to help workers adapt their circadian rhythm to work at night and sleep during the day although such activity schedules were not investigated in this manuscript. Currently, light-based interventions are being explored to improve shift worker’s performance and health.^33^

### 5.2 ipRGC-influenced health evaluation comparison

Finding a balance between definitive evaluations (i.e. pass-fail health design metrics) and simulating nuanced information about the biological effects of lighting design is challenging. On one hand, this study demonstrates how existing metrics and previous approaches may not fully capture light’s influence on ipRGC-influenced effects. Phase shifting, subjective sleepiness and melatonin suppression do not respond the same way to space design and operation changes, and phase shifting is not correlated with any existing ipRGC-influenced lighting metric. This concept is also demonstrated by the varying correlation strengths between nvRD, N-VE, the WELL standard and other threshold metrics with the iNSOM-simulated^34^ photobiological symptoms. These findings are sensible because the circadian system is complex with many different symptoms that respond to light. A singular circadian health design metric may not be appropriate. For example, in the photobiological model implemented here,^1–3^ light’s instantaneous effect on subjective sleepiness (KSS) is quickly saturated compared to melatonin suppression. We hypothesize that the low threshold for KSS saturation compared to the relatively high threshold to meet most daytime standards explains why they are only practically correlated with KSS during morning and afternoon periods. Such variability between ipRGC-influenced effects may limit the applicability of metrics that determine the likelihood of a general ipRGC-influenced response rather than towards each ipRGC-influenced outcome.

Due to the circadian system’s sensitivity to timing, categorizing the day into distinct periods may be a step towards circadian health design metrics that better relate to photobiological symptoms.^16,17^ For example, the nighttime Brown *et al*.^15^ recommendation is strongly correlated with melatonin suppression, because melatonin production primarily occurs during the evening period. Compared to the other metrics, Brown *et al*.’s^15^ thresholds’ clear relationship with melatonin suppression (morning, evening), KSS (especially evening) and vPVTRT suggests that it may be an effective simplification for evaluative purposes. On the other hand, N-VE avoidance seems to best identify changes in vPVTRT reaction time. All existing metrics that we compared had meaningful correlations with many photobiological outcomes. Evaluative metrics are still a work in progress.

In practice, simulating biological symptoms grants a more nuanced understanding of a building’s effect on a human occupant’s circadian system and may allow designers to make special considerations depending on the programme of the project. For example, designers may focus on alertness for an office building or proper circadian entrainment in a project with long-term occupancy such as a healthcare facility, home or penitentiary.

### 5.3 Limitations

Despite the potential for the model to be applied for the design of healthy circadian lighting, there are some limitations to consider. Firstly, the photobiological model in this paper is based on highly controlled medical experiments that do not mirror a realistic sleep–wake cycle. By adding in other highly differing variables like daylight, electric light controls and occupant routine changes (scheduled or disrupted sleep, screen use) to a model that was created under very rigid conditions, we cannot be sure that the model is reacting realistically to the inputs. Future work is needed to test and validate ipRGC-influenced metrics for people functioning in their natural environment and routines. This also represents a challenge for software developers – how should the stark differences in light exposure to different behavioural aspects illustrated in this paper (sleep, lighting controls) be presented within a design? And how should aspects like age and chronotype be included in the future? In programmes where occupants are transient, the applicability of iNSOM for design becomes even more challenging and may require further research on how people experience light outside of working hours, for example.

As with iNSOM’s core photobiological model, the metrics compared against it have not been validated using human photobiological response data from outside of the lab. Thus, the models were discussed in relation to each other, and their accuracy may only be discussed speculatively based on previous research. This limitation is further highlighted by one study in the sparse validation literature that demonstrated large prediction errors between measured alertness and alertness predicted by photobiological models including Tekieh *et al*.^3^ and Amundadottir *et al*.^12^ discussed in this paper.^32^ Future research needs to validate the current lighting and circadian health metrics using measured light and photobiological response data in realistic settings.

The iNSOM metrics are also descriptive rather than evaluative. While there is some direction on what is the preferred occupant circadian outcomes such as minimizing melatonin suppression in the evening, future work is needed to determine precisely how much alertness, phase shifting and melatonin suppression is optimal to translate the photobiologically derived metrics into binary measures useful for evaluating architectural design. For now, the Brown *et al*.^18^ thresholds provide reasonable and concrete values based on scientific consensus to target as a designer.

## 6. Conclusion

Despite its limitations and required future work, iNSOM demonstrates a significant step forward for evaluating ipRGC-influenced lighting design. The photobiologically derived metrics are annualized and directly indicate predicted symptoms on the ipRGC-influenced system (KSS, reaction time, melatonin suppression and circadian phase shifting) as an occupant would experience them. Directly predicting ipRGC-influenced metrics offers designers meaningful data that gets to the root purpose of ipRGC-influenced lighting design and highlights how impactful architecture can be on occupant health.

## Supplemental Material

sj-docx-1-lrt-10.1177_14771535251368380 – Supplemental material for An ipRGC-influenced/Non-Visual Spectral Occupant Model for lighting design, Part 2: Photobiological model implementation Supplemental material, sj-docx-1-lrt-10.1177_14771535251368380 for An ipRGC-influenced/Non-Visual Spectral Occupant Model for lighting design, Part 2: Photobiological model implementation by J Alstan Jakubiec and A Alight in Lighting Research & Technology

## Appendix

**Appendix A:** Terminology, metrics and measures

Terms	Definition
Brown *et al*.^15^ recommendations	Recommended thresholds developed at the Second International Workshop on Circadian and Neurophysiological Photometry in 2019 to support physiology, sleep and wakefulness in healthy adults using SI standard units of melanopic EDI ( $E_{v,\mathrm{mel}}^{D65}$ ). • Daytime: $E_{v,\mathrm{mel}}^{D65}>250\;\mathrm{lx}$ • Three hours before sleep: $E_{v,\mathrm{mel}}^{D65}<10\;\mathrm{lx}$ • During sleep: $E_{v,\mathrm{mel}}^{D65}<1\;\mathrm{lx}$
*C* ^1^	The circadian drive as calculated by the Postnova *et al*. model of arousal and light dynamics. *C* is periodic across an approximately 24-h time scale. As *C* grows, it inhibits sleepiness and is modulated by sleep history and light exposure.
*E*_e,mel_^22^	SI quantity of melanopic irradiance in units of W m^−2^– weighted irradiance as per the CIE S 026 system. *E*_e,mel_ of 1 W m^−2^ is equivalent to $E_{v,\mathrm{mel}}^{D65}$ of 754 lx.
$E_{v,\mathrm{mel}}^{D65}$ ^22^	SI quantity of melanopic EDI in units of lx. $E_{v,\mathrm{mel}}^{D65}$ is the quantity of D65 spectrum illuminance needed to produce the same melanopic impact as the light being characterized. $E_{v,\mathrm{mel}}^{D65}$ of 250 lx is equivalent to *E*_e,mel_ of 0.22 W m^−2^.
EML	Equivalent melanopic lux, an outdated measure of melanopic light still used in industry and some standards in units of lx. EML of 250 lx is equivalent to $E_{v,\;\mathrm{mel}}^{D65}$ of 227 lx.
*H* ^1^	The homeostatic drive as calculated by the Postnova *et al*. model of arousal and light dynamics. *H* increases during wake and decreases during sleep. As *H* increases, it drives sleepiness.
KSS^29^	The Karolinska Sleepiness Scale, a 9-point subjective rating system for sleepiness. For example, • 1: Extremely alert • 5: Neither alert not sleepy • 9: Very sleepy, fighting sleep
Melatonin suppression^2,3^	The percentage of melatonin concentration in the blood suppressed due to exposure to light as compared against the absence of light exposure and evaluated using integrals of melatonin concentration over time.
N-VE^16,17^	An assessment framework for ‘non-visual effects’ due to light exposure, originally developed for daylight alone. 960 D55 spectrum equivalent lux are required for a full ipRGC-influenced lighting response, and levels below 210 D55 equivalent lux are determined to not have an ipRGC-influenced effect. N-VE is evaluated across three time periods which are also descriptively named, • Circadian resetting: 6.00 to 10.00 • Alerting: 10.00 to 18.00 • Light avoidance: 18.00 to 6.00
nvRD^12^	Non-visual direct response, a cumulative daily light exposure framework that targets a result of 4.2 units based on receiving 824 D65-equivalent lx for a period of 5 h or greater each day.
Phase shift^1^	The cumulative difference in peak melatonin blood plasma concentration over a period of time. In this manuscript, seasonally and reported in minutes (min).
vPVTRT^1^	Response time in milliseconds (ms) on a visual performance vigilance test.
WELL standard^13^	An often-changing standard for ‘circadian lighting design’. The WELL standard, at the time of this paper’s evaluation, required EML > 250 lx on eyes in daytime working spaces and EML < 50 lx on workplanes in residential spaces at night.
