# Supplementary material for: An ipRGC-influenced/Non-Visual Spectral Occupant Model for lighting design, Part 2: Photobiological model implementation
Source: Light Res Technol. 2025 Oct 14;58(1-2):23–46. doi: 10.1177/14771535251368380 (PMC12994914; doi:10.1177/14771535251368380)
Supplement: sj-docx-1-lrt-10.1177_14771535251368380 – Supplemental material for An ipRGC-influenced/Non-Visual Spectral Occupant Model for lighting design, Part 2: Photobiological model implementation [file sj-docx-1-lrt-10.1177_14771535251368380.docx]

Supplemental information: photobiological model equations

# An ipRGC-influenced/non-visual spectral occupant model (iNSOM) for lighting design, part 2: photobiological model implementation

**J. Alstan Jakubiec**^1,2^ and **Athina Alight**^1^

^1^ John H. Daniels Faculty of Architecture, Landscape and Design, University of Toronto, Toronto, Canada

^2^ School of the Environment, University of Toronto, Toronto, Canada

Herein are the equations used in our photobiological model implemented following Postnova, et al.;^1^ Abeysuriya, et al.;^2^ and Tekieh, et al.^3^

# Model Equations

(S1)^1^ $dV_{v}$, change in voltage of sleep-active neurons (VLPO)

$t_{v}\frac{{dV}_{v}}{dt}= V_{vm}\cdot Q_{m}-V_{v}+D_{v}$

where,

$t_{v}=50 s$

$v_{vm}= -2.1 mV$

(S2)^1^ ${dV}_{m}$, change in voltage of wake-active nuclei (MA)

$t_{m}\frac{dV_{m}}{dt}=v_{mv} \cdot Q_{v}-V_{m}+D_{m}+W$

where,

$t_{m}=50 s$

$v_{mv}= -1.8 mV$

$D_{m}=1.3 mV$

(S3)^1^ $dH$, change in homeostatic drive

$t_{H}\frac{dH}{dt}=v_{Hm}\cdot Q_{m}-H$

where,

$t_{H}= 212400 s$

$v_{Hm}=4.57 s$

(S4)^1^ $dX$, change in circadian variable ‘X’

$t_{X}\frac{dX}{dt}=Y+\gamma\cdot\left( \frac{X}{3}+\frac{4X^{3}}{3}+\frac{256X^{7}}{105} \right)+v_{Xp}\cdot D_{p}+v_{Xn}\cdot D_{n}$

where,

$t_{X}= 43200/\pi$ s

$\gamma=0.13$

$v_{Xp}=2220 s$

$v_{Xn}=0.032$

(S5)^1^ $dY$, change in circadian variable ‘Y’

$t_{Y}\frac{dY}{dt}=D_{p}\cdot\left( v_{YY}\cdot Y-v_{YX}\cdot X \right)-\left( \frac{d}{t_{C}} \right)^{2}\cdot X$

where,

$t_{Y}= 43200/\pi$ s

$v_{YY}=730 s$

$v_{YX}=1221 s$

$d=\frac{86400}{0.99729}s$

(S6)^1^ $dP$, change in photoreceptor activity

$\frac{dP}{dt}=\propto\cdot\left( 1-P \right)-\beta\cdot P$

where,

$\beta=\frac{0.007}{60}$ s^-1^

(S7)^1^ $Q_{i}$, mean population firing rate ($Q_{v}$ for VLPO and $Q_{m}$ for MA)

$Q_{i}=\frac{Q_{max}}{1+\exp\left( \frac{\theta-V_{i}}{\sigma^{'}} \right)}$

where,

$Q_{\max} =100$s^-1^

$\theta=10 mV$

$\sigma^{'}=3 mV$

(S8)^1^ $W$, wake effort

$W=F_{w}\cdot max\left( 0, V_{WE}-v_{mv}\cdot Q_{v}-D_{m} \right)$

where,

$F_{w}=1$ if forced wake, otherwise 0

$V_{WE}= -0.07$ mV

$v_{mv}= -1.8 mV$

$D_{m}=1.3 mV$

(S9)^1^ $D_{v}$, drive to sleep-active neurons (VLPO)

$D_{v}=v_{vH}\cdot H+v_{vC}\cdot C+A_{v}$

where,

$v_{vH}=1$

$v_{vC}= -0.5$ mV

$A_{v}= -10.3 mV$

(S10)^1^ $C$, circadian drive

$C=0.1\cdot\frac{1+X}{2}$ + $\left( \frac{3.1\cdot X-2.5\cdot Y+4.2}{3.7\cdot\left( X+2 \right)} \right)^{2}$

(S11)^1^ $D_{n}$, nonphotic drive to the circadian

$D_{n}=\left( S-\frac{2}{3} \right)\cdot(1-\tanh\left( r\cdot X \right))$

where,

$r=10$

(S12)^1^ $D_{p}$, photic drive to the circadian

$D_{p}= \propto\cdot(1-P)\cdot(1-\varepsilon\cdot X)\cdot(1-\varepsilon\cdot Y)$

where,

$\varepsilon=0.4$

(S13)^1,3^ $\propto$, photic response

$\propto= \propto_{0}\cdot\left( \frac{I}{I+I_{1}*F_{4100K}} \right)\cdot\sqrt{\frac{I}{I_{0}*F_{4100K}}}$

where,

$\propto_{0}=\frac{0.1}{60}$ s

$I=$ melanopic irradiance in W/m^2^

$I_{0}=100 lx$

$I_{1}=9500 lx$

$F_{4100K}= 0.00080854$ W/m^2^/lx

(S14)^1^ $S$, sleep / wake state

if scheduled wake or $V_{m}>V_{th},$

$S=1$ (wake)

else,

$S=0$ (sleep)

(S15)^3^ $r$, melatonin suppression

$r=1-\frac{r_{a}}{1+\left( \frac{I}{r_{b}} \right)^{-r_{c}}}$

where,

$I=$ melanopic irradiance in W/m^2^

$r_{a}$ = 1.0

$r_{b}$ = 0.031

$r_{c}=0.82$

(S16)^2,3^ $dAt$, change in melatonin synthesis

$t_{A}\frac{dAt}{dt}=m\cdot r-At$

where,

$t_{A}=$ 5400 s

if $atan2(Y,X)<-1.44$ or $atan2(Y,X)>2.78$,

$m=1$

else,

$m=0$

(S17)^2^ $dp_{b}$, change in melatonin blood plasma concentration

$\frac{dp_{b}}{dt}=\frac{u^{*}At}{r_{g}}-u^{*}\cdot\frac{p_{b}}{r_{g}\cdot p_{b}^{*}}$

where,

$u^{*}=$ 0.47 pmolL^-1^s^-1^

$p_{b}^{*}=325.0$ pmolL^-1^

$r_{g}$= 0.9

(S18)^3^ $S$, instantaneous alerting effect of light

$S(I)=\frac{1}{1+\exp\left( \frac{S_{b}-I}{S_{c}} \right)}$

$S_{norm}=\frac{S\left( I \right)-S\left( 0 \right)}{S\left( 1000 \right)-S(0)}$

where,

$I=$ melanopic irradiance in W/m^2^

$S_{b}=$ 0.05 W/m^2^

$S_{c}=$ 1 / 223.5 m^2^/W

(Note that the published version of $S_{c}$ is 223.5 but we use 1/223.5

following an e-mail correspondence with Dr. Tekieh and Dr. Postnova.)

# Metric Equations

(S19)^3^ $d\theta_{L}$, change in homeostatic alerting effects due to light

$t_{L}\frac{d\theta_{L}}{dt}=$-$\theta_{L}+v_{LA}\cdot S_{norm}$

where,

$t_{L}=1440$ s

$v_{LA}=-0.11$

(S20)^1,3^ $KSS$, Karolinska sleepiness scale prediction

$KSS=c_{KSS}+\left( \theta H_{KSS}+ \theta_{L} \right)\cdot H+ \theta C_{KSS}\cdot C$

where,

$c_{KSS}=-24.34$

$\theta H_{KSS}$ = 2.28

$\theta C_{KSS}= -1.74$

(S21)^1^ $vPVTRT$, predicted mean reaction time on a visual performance vigilance test

$vPVTRT_{mean}=c_{vPVTRT-mean}+\theta H_{vPVTRT-mean}\cdot H+ \theta C_{vPVTRT-mean}\cdot C$

where,

$c_{vPVTRT-mean}=-12787$ ms

$\theta H_{vPVTRT-mean}=1055$

$\theta C_{vPVTRT-mean}= -1144$

# Starting Values

The starting dynamic values we use are as follows.

| $p_{b}, p_{bmax}=200$ | Estimated from Figure 2, Abeysuriya, et al.^2^ |
| --- | --- |
| $At,At_{max} =\frac{0.47}{2.0}$ | Estimated from Table 1, Abeysuriya, et al.^2^ |
| $\theta_{L}=0$ | The model begins at midnight with no irradiance present. |
| $V_{v}=1.5$ | Estimated from Figure 5, Phillips and Robinson.^4^ |
| $V_{m}=-15.0$ | Estimated from Figure 2, Postnova, et al.^5^ |
| $H=13$ | Estimated from Figure 2, Postnova, et al.^5^ |
| $X=0.04$ | Estimated from St. Hilaire, et al.^6^ |
| $Y= -1.28$ | Estimated based on values of X = 0 and C = 1 from Postnova, et al.^7^ |
| $P=0$ | Assume no photoreceptors are firing during sleep. |
| $C=1$ | From Postnova, et al.^7^ |

# References

1. Postnova S, Lockley SW, Robinson PA. Prediction of cognitive performance and subjective sleepiness using a model of arousal dynamics. Journal of Biological Rhythms 2018; 33(2): 203–18.
2. Abeysuriya RG, Lockley SW, Robinson PA, et al. A unified model of melatonin, 6-sulfatoxymelatonin, and sleep dynamics. Journal of Pineal Resesarch 2018; 64(4): e12474.
3. Tekieh T, Lockley SW, Robinson PA, et al. Modeling melanopsin-mediated effects of light on circadian phase, melatonin suppression, and subjective sleepiness. Journal of Pineal Research 2020; 69(3): e12681.
4. Phillips AJ, Robinson PA. A quantitative model of sleep-wake dynamics based on the physiology of the brainstem ascending arousal system. Journal of Biological Rhythms 2007; 22(2): 167-79.
5. Postnova S, Postnov DD, Seneviratne M, et al. Effects of rotation interval on sleepiness and circadian dynamics on forward rotating 3-shift systems. Journal of Biological Rhythms 2014; 29(1): 60-70.
6. St. Hilaire MA, Klerman EB, Khalsa SBS, et al. Addition of a non-photic component to a light-based mathematical model of the human circadian pacemaker. Journal of Theoretical Biology 2007; 247(4): 583–99.
7. Postnova S, Lockley SW, Robinson PA. Sleep propensity under forced desynchrony in a model of arousal state dynamics. Journal of Biological Rhythms. 2016; 31(5): 498-508.
